# Strategies to Enhance Seasonal Influenza Vaccination Uptake: Qualitative Insights from Primary Care Physicians in Greece

**DOI:** 10.3390/vaccines14050458

**Published:** 2026-05-20

**Authors:** Ilias Pagkozidis, Georgios Papazisis, Anna-Bettina Haidich, Zoi Tsimtsiou

**Affiliations:** 1 Department of Hygiene, Social-Preventive Medicine and Medical Statistics, School of Medicine, Aristotle University of Thessaloniki, 54124 Thessaloniki, Greece; pagoilias@gmail.com (I.P.); haidich@auth.gr (A.-B.H.); 2 Department of Clinical Pharmacology, School of Medicine, Aristotle University of Thessaloniki, 54124 Thessaloniki, Greece; papazisg@auth.gr

**Keywords:** seasonal influenza vaccination, primary care, physicians, vaccine confidence, vaccine uptake, qualitative study, Greece

## Abstract

**Background/Objectives**: Primary Care Physicians (PCPs) are widely regarded as trusted sources of health information and can play a pivotal role in increasing seasonal influenza vaccination (SIV) within their communities. We aimed to explore PCPs’ attitudes toward SIV and their views regarding proposed strategies to enhance SIV uptake in the evolving post-pandemic landscape. **Methods**: A qualitative study utilizing semi-structured individual interviews with a nationwide sample of 25 PCPs was conducted. **Results**: Physicians’ attitudes toward SIV were overwhelmingly positive; they recognized its protective value for individuals and the community alike, its efficacy in averting serious illness, and its proven safety profile. Regarding strengthening SIV uptake, PCPs positively appraised the following strategies: (a) viewing all clinical encounters as opportunities for vaccination; (b) outsourcing vaccination to nursing, allied health staff and community pharmacists, provided that specific prerequisites are met; (c) forwarding personalized notifications to health providers and (d) the public; and (e) establishing at-home vaccinations. Financial incentives would reportedly act as tangible acknowledgement and motivate PCPs to work toward primary prevention. However, others have argued that SIV is inherently embedded in their duty as PCPs, and potential remunerations would dwindle the public’s confidence in PCPs. Establishing incentives for the general population reportedly minimizes confidence and the perceived value of SIVs and was assessed to be ineffective in the Greek context. Promoting SIVs through video games was considered to be less effective for the adult population. **Conclusions**: Mapping PCPs’ insights is key in designing effective SIV strategies that are concurrent with communities’ values, needs, and learnt experience from the COVID-19 pandemic.

## 1. Introduction

Seasonal influenza is an acute respiratory infection with considerable burden for the general population and health systems alike. At present, the World Health Organization estimates that around one billion seasonal influenza cases are annually diagnosed, while accounting for around five million severe illnesses and 290,000 to 650,000 annual deaths [1]. Adults over the age of 65 are disproportionately affected by seasonal influenza, with the highest hospitalization and mortality rates being recorded within this demographic group [2]. Healthcare costs associated with seasonal influenza skyrocket during peak illness periods, and nosocomial outbreaks add more than €100.000 in direct and indirect costs [3]. In the US, the economic impact of influenza in healthcare was calculated at as high as $11 billion [4]. Increased absenteeism from work and school environments is also noted during peak periods and leads to productivity losses, which are estimated at around €10 billion annually [5].

Immunization has long been acknowledged as one of the most successful and efficient public health interventions. It constitutes the single most effective way to prevent and reduce the burden of seasonal influenza. The Centers for Disease Control and Prevention (CDC) estimates that seasonal influenza vaccination (SIV) has averted around 11 million illnesses, five million flu-related medical visits and 12 thousand deaths [2]. SIV averted around two fifths of hospitalizations among individuals over the age of 65 during the 2024/2025 season. Countries with higher vaccination coverage yielded greater benefits [6]. Nevertheless, SIV coverage among adults, especially the elderly and those belonging to high-risk groups, remains insufficient. Most EU/EEA countries have reported a recent decreased and inadequate SIV uptake compared to former seasons [7]. Only four countries approached the 75% recommendation target for SIV among the adult population during the 2024/25 season, an objective that was met in the US according to available data [8].

With regard to national data, a recent nationwide study estimated SIV coverage to be around 55% among individuals belonging to high-risk groups in Greece [9]. Kossyva et al. reported that around four fifths of the population in a Primary Health Care (PHC) unit in Crete were covered for influenza during the 2024/5 season [10]. Avramidis et al. also reported on the high SIV coverage among adults in a region of Northern Greece, with more than three quarters of study participants having been vaccinated in the 2021/2 season [11]. Under the Greek National Health System and related vaccination policies, SIV is recommended and fully reimbursed for individuals ≥60 years old, and for adults belonging to vulnerable risk groups. Adjuvant and high-dose vaccines are also available and recommended for those ≥65 years old. For the 2025/6 season, vaccine delivery pathways had been modified, with community pharmacists allowed to consult, prescribe and administer SIV without direct oversight and the need for prescription from PCPs. However, immunization with adjuvant and high-dose vaccines still required prescription from PCPs. Following the intense, multi-dose COVID-19 vaccination campaigns, vaccine fatigue has emerged among the general population, manifesting in the form of negligence and aversion to getting immunized [10].

Recommendations and advice from healthcare workers are among the strongest factors that increase the general population’s confidence in vaccines and positively influence their intention to receive vaccinations [12,13,14]. Contact and consultation with healthcare providers are crucial in instilling awareness of SIV recommendations among the patient population. Through patient–PCP discussions, negative perceptions regarding SIV can be overturned, especially among high-risk groups and the elderly [14]. Recent studies underscored that Primary Care Providers (PCPs) constituted the key informants on SIV and that patients are up to 11 times more likely to get vaccinated against influenza following their provider’s or pharmacist’s recommendation [9,11]. Indeed, PCPs are perceived as trusted sources of information and health advisors. They serve as key actors in increasing SIV awareness within communities, and aid in minimizing hesitancy and reinstating confidence in immunizations [15].

Despite having showcased increased awareness over the importance and the need to prioritize SIV [16], post-pandemic vaccine fatigue and the unique nature of the annual SIV represent a key challenge for PCPs. Within this unique, post-COVID-19 landscape, we aimed to explore attitudes toward SIV through the lens of frontline PCPs in the Greek healthcare context. This article further maps out the actionable strategies to bolster SIV uptake among the general population, as evaluated by PCPs. Highlighting PCPs’ perceptions and proposals are key in understanding the potential drivers, hesitation and barriers regarding SIV recommendation.

## 2. Materials and Methods

### 2.1. Study Population and Sampling

To better understand and analyze PCPs’ attitudes and their suggestions regarding strategies to enhance adult vaccination in the post-COVID 19 era, a qualitative study was employed. PCPs from across Greece were invited to take part in semi-structured, individual interviews. The latter were conducted during January–June 2025. Study participants met the following criteria: (a) practicing as General Practice/Family Medicine (GP/FM) physicians; and (b) employed in primary care units, either public or private, during the study period. To maximize outreach, an email invitation was sent to members of a GP/FM association. Participating PCPs were additionally asked to forward the study invitation to their professional networks. Purposive and snowball sampling were employed to better capture the qualitative insights of female and male PCPs. Also, participating PCPs practiced across a diverse range of geographic areas, including urban, semi-urban and rural settings in both mainland Greece and the islands. The patient population is thus diverse and may vary in terms of key demographics. PCPs practicing in rural settings mainly cater to older, geographically isolated populations. Conversely, in semi-urban and densely populated urban settings on the mainland, PCPs cater for a heterogenous patient population. Recruitment was conducted simultaneously with data analysis and was discontinued once data saturation was achieved. No incentives were provided to study participants. PCPs were informed of their right to withdraw their consent and have their data removed without any repercussions. No dropouts, repeat and follow-up interviews were recorded.

### 2.2. Data Collection and Analysis

The semi-structured interview guide was developed based on extensive literature review and in consultation with experts. Pilot interviews were initially conducted with three PCPs to evaluate the relevance, clarity, and flow of the interview guide. The semi-structured guide was reviewed based on the feedback from (a) the pilot interviews; and (b) an additional independent researcher with experience in qualitative methodology. The guide was subsequently adjusted to increase the neutrality of phrasing, reduce ambiguities, and strengthen comprehensibility and the flow of conversation. As the interview guide was eventually modified, the data from the three pilot interviews were discarded from the final analysis to ensure consistency, and the final interview guide was developed. Findings on the impact of the COVID-19 pandemic on the public’s attitudes and stances toward adult vaccination, as perceived by frontline PCPs, as well as their suggestions on building on vaccine confidence that derived from a part of this interview guide have already been published [17]. This article reports on the qualitative insights of PCPs regarding their attitudes toward SIV, and their appraisal of suggested strategies to bolster SIV uptake among the adult population. The part of the interview guide relevant to the presented findings is presented in Supplementary File S1. Interviews were conducted outside of office hours by the first author. They were conducted remotely, via Zoom and Microsoft Teams, and were audio recorded and transcribed verbatim. Anonymization of identifiable data was ensured. Field notes were taken during the interviews and were used, alongside the recordings, to check for inconsistencies and to increase the accuracy of transcripts.

Thematic analysis was conducted to analyze raw data provided by the semi-structured interviews [18]. Transcribing and data analysis were performed independently by two researchers (IP and SD), PCPs with experience in qualitative methodology. Concepts and themes were identified and formulated from available raw data, utilizing an inductive approach [19]. Researchers initially immersed themselves in the first available set of transcripts, identified recurring themes, and developed preliminary codes. They then conducted a thorough, line-by-line reading of transcripts and identified interesting aspects in the available data. They open-coded transcripts and dynamically created new codes, as novel concepts emerged during the analysis. They also extracted data and broader text segments to preserve participants’ original context relevant to each code. Preliminary overarching themes were developed and reviewed to evaluate fitness to the respective transcripts. To ensure the trustworthiness of our analysis, a consensus-building approach was utilized. Following the independent coding of transcripts, the research team then met to compare themes and codes and discuss differences in interpretation and coding. Divergent interpretations were viewed as opportunities for deeper analytical reflection. A third independent researcher (ZT) acted as a validator during these meetings by facilitating reflexive discussions, exploring and bridging divergent views and refining the coding and thematic framework. The common coding framework was agreed upon during these meetings, was applied in available and future transcripts and was iteratively refined.

Participant recruitment was concluded once data saturation was reached. To determine data saturation, data analysis was performed simultaneously with PCP recruitment [20]. For the purposes of this study, as per the Hennink et al. framework [21], we ceased participant recruitment once no new codes (coding saturation) and insights into existing codes and themes (meaning saturation) materialized in the last three transcripts. After the initial 12 interviews, subsequent transcripts were mapped against the coding framework to check for the emergence of novel codes and insights. By interview 22, no new codes or themes were brought up. Recruiting three more PCPs confirmed coding and meaning saturation in our sample, as they yielded insights that reflected those in the already established themes and categories from our analysis. The research team followed the Standards for Reporting Qualitative Research (SRQR) for the design, implementation and reporting of findings [22].

A bracketing approach was followed to reduce researchers’ personal bias. The researchers involved in study design, data collection, analysis and reporting are vaccine supporters and are eager to strengthen the vaccine confidence and uptake of their communities. Before commencing the study, researchers acknowledged how their personal values, beliefs and inherent biases may intersect with the studied phenomenon. The interview guide was thoroughly revised by an independent researcher, a non-healthcare worker who is experienced in qualitative methodology. Utilizing the feedback provided, the interview guide was revised to enhance neutrality of phrasing to reduce socially desirable responding. During the semi-structured interviews, the primary researcher remained aware of his own beliefs on the issue, set aside personal preconceptions, and maintained an impartial stance. Abiding by the neutral and open-ended phrasing of the interview guide, he ensured that PCPs were not led towards specific answers and delved into PCPs’ opposing views. The a priori established unfamiliarity with the research team also minimized the social desirability bias of study participants and maximized the documentation of truthful responses. During the data analysis, personal bias was minimized. Researchers independently coded the available transcripts, and consensus meetings were held to compare and defend codes and themes using extracts and context from the raw data. The evident content of available data was interpreted and themes were iteratively compared against the raw transcript extracts. This ensured that the final thematic framework authentically reflected PCPs’ views, did not de-emphasize opposite stances, and was not influenced by researchers’ personal standpoints.

## 3. Results

Twenty-five PCPs participated in the study (60% women, *n* = 15), with a mean age of 44.9 years (SD 8.3, min. 32–max. 62). Regarding their years of experience in PHC settings, three distinct groups were noted: (a) 0–5 years (32%); (b) 5–15 years (36%); and (c) more than 15 years (32%). They practiced in 11 different prefectures within 6 geographic regions in Greece, namely Attica (40%), Thessaly (16%), Central Macedonia (16%), Eastern Macedonia and Thrace (8%), Western Macedonia (8%), Western Greece (8%), and North Aegean (4%). Eleven (44%) PCPs practiced in urban, five (20%) in semi-urban and nine (36%) in rural areas. The demographic and professional characteristics of study participants are illustrated in Table 1.

### 3.1. PCPs’ Attitudes Toward Seasonal Influenza Vaccination

SIV was perceived positively by all participating PCPs. They primarily lauded its value, effectiveness and safety profile, which proved itself over the course of time. SIV was reportedly imperative for those in contact with vulnerable individuals, as it provides invaluable protection for patients with comorbidities and communities alike. SIV also curtails the economic burden of absenteeism from work during peak flu seasons. Participants highlighted SIV’s effectiveness in preventing illness and reducing the burden on health systems by minimizing complications, averting hospitalizations and death. Few PCPs cited the inaccuracy and the low potency of SIV in past seasons. This finding did not discourage them, though, from recommending the vaccine in the following seasons. Despite the reported negligence and doubts over the novel, high-dose and adjuvanted vaccines, SIV was widely accepted by the general population. PCPs underscored the ease in persuading patients, due to individuals’ immunization-seeking behavior and the prevalent notion that SIV is annually mandated. SIV was broadly accepted by PCPs who easily engaged in relevant conversations with patients and were willing to recommend and prioritize this vaccine, even to individuals that do not meet the reimbursement criteria. Two PCPs proposed lowering the age criterion for reimbursement of SIV and introducing compulsory SIV for elder citizens. Main themes and selected illustrative quotes regarding attitudes toward SIV are presented in Table 2.

### 3.2. Strategies to Strengthen Seasonal Influenza Vaccination

PCPs in our study commented on strategies considered vital in bolstering SIV [23,24,25,26,27,28,29,30,31,32,33,34,35,36,37,38,39,40]. The eight main strategies discussed were: (a) viewing all clinical encounters as opportunities for SIV; (b) outsourcing vaccination to nursing, allied health workers and community pharmacists; (c) the provision of personal notifications to the public about SIV; (d) the provision of personal notifications to health providers about SIV; (e) the adoption of incentives for the general population; (f) the establishment of incentives for health providers; (g) the establishment of at-home vaccination programs; and (h) raising SIV awareness through gamification. The main themes and selected illustrative quotes regarding participants’ views toward these strategies are presented in Table 3.

#### 3.2.1. Opportunistic Counseling Regarding SIV at All Clinical Encounters

Participants perceived patient counseling at every clinical encounter during the SIV period to be part of their duty as PCPs. Though on-site vaccination may not be currently feasible within the Greek context, PCPs noted that recommendations of SIV remained pivotal in outpatient appointments and emergency settings, depending on the clinical context. During peak seasons, they commented further on the need for a holistic approach, and to boost protection and make the most out of each patient encounter by offering advice and suggesting SIV to relatives and chaperones as well. Elongating appointments, strengthening patients’ health literacy and utilizing the patient–PCP bond and trusted relationship, were deemed crucial in the successful advocacy for SIV.

Representing a divergent view in our sample, five PCPs opposed the notion of opportunistic counseling, citing (a) a lack of available time during consultations to address SIV and approach the vaccination status of family members; (b) the resistance of chaperones; and (c) PCPs’ need to avoid conflict with individuals whose consultation agenda did not include SIV (Table 3).

#### 3.2.2. Outsourcing Vaccination to Nursing, Allied Health Workers and Community Pharmacists

Delegating vaccinations to diverse health worker disciplines was deemed effective in the effort to increase SIV uptake. PCPs valued the importance of strengthening the role of non-physician health workers in SIV within the Greek PHC context and community pharmacies.

Outsourcing vaccination capabilities was considered effective in boosting SIV coverage on condition that: (a) allied health workers were offered access and input capabilities in the SIV registry; (b) allied health workers were trained to properly perform immunizations and combat potential complications; (c) the National Immunization Program criteria and contra-indications were thoroughly checked before vaccinating; and (d) PCPs remained the focal point of contact and oversaw the process of SIV.

Conversely, eight PCPs preferred SIV to remain under the direct control of PCPs, citing (a) negative prior experience; (b) reported errors in dispensaries; and (c) the need to own responsibility for patients’ health and any subsequent medical procedures (Table 3).

#### 3.2.3. Notifications Regarding SIV to the General Population

Notifying the general population about the flu and SIV via email and/or text messages was considered beneficial in boosting uptake. Following in the footsteps of the recently established nationwide alerts for colon, breast, and cervical cancer and for cardiovascular disease screening, PCPs proposed expanding the notifications for primary prevention campaigns as well. Reminders would reportedly raise awareness and empower citizens to take responsibility for their own health. Utilization of personalized messages and reminders to consult with their local PHC units and strong political will were key in the successful expansion of the already-tested format.

Diverging from the predominant sentiment, personalized reminders were deemed not feasible by four PCPs. They noted issues of low levels of digital literacy among the public, the exclusion of older citizens, and a lack of initiating meaningful action from patients’ side, as drawn from past experience (Table 3).

#### 3.2.4. Notifications Regarding SIV to PCPs

The establishment of reminders targeted towards PCPs was unanimously deemed an effective strategy to increase SIV uptake and coverage. Notifications reportedly compliment and facilitate PCPs’ everyday workload. They act as a safety mechanism for overburdened healthcare professionals to not neglect annual discussions with patients on the matter of vaccination against the flu. To successfully establish and run a notification system for PCPs, four main prerequisites were underscored, including the establishment of a vaccine registry, PCPs’ training and awareness thereof, and elongating PHC appointments. With regard to the reminders’ format, participants preferred to be notified prior to commencing the appointment to increase preparedness for SIV discussions. The notification should ideally be placed within the interface of the electronic medical record, avoiding pop-up messages that reportedly tend to be ignored (Table 3).

#### 3.2.5. Adoption of Incentives for the General Population

Most participants were opposed to establishing incentives targeted at encouraging the general public to get SIV. Drawing from prior negative experience with compulsory measures to increase the COVID-19 vaccine uptake, provision of financial remuneration for SIV was deemed ineffective in the Greek context. PCPs considered incentives to minimize the value of primary prevention and dwindle the public’s confidence in vaccinations. They strengthen conspiracy thinking and hesitancy and have a spillover effect on childhood immunizations.

Offering contrasting perspectives, two PCPs proposed the adoption of deductions in medications and laboratory examinations for those vaccinated against the flu. Ultimately, in an effort to boost SIV uptake, one PCP suggested that unvaccinated individuals co-pay their potential hospitalization costs (Table 3).

#### 3.2.6. Adoption of Incentives for PCPs

PCPs were divided over the value of establishing incentives for PCPs to increase SIV uptake. Financial remuneration or deductions in educational programs and lifelong learning activities were considered a form of recognition of PCPs’ work in primary prevention. Drawing from personal experience with other prevention schemes, participants considered remuneration to increase compliance and motivate colleagues in their efforts to communicate SIV.

However, most PCPs were opposed to the provision of incentives, as primary prevention and SIV were deemed a core care duty for PHC providers. The commercialized nature of establishing remuneration schemes for SIV uptake would also reportedly increase resentment across the general population, further dwindling confidence and trust in vaccines and PCPs alike (Table 3).

#### 3.2.7. Establishment of At-Home Vaccination Programs

PCPs lauded the invaluable importance of organized, at-home vaccination programs for frail, multimorbid patients and those unable to reach PHC units. Protecting susceptible individuals from the flu would aid in preventing outbreaks in residential settings and reduce the burden of healthcare systems. Participants called authorities to establish organized, mobile vaccination schemes, drawing from the experience and outcomes of the COVID-19 at-home vaccination program and the mass tetanus vaccinations following natural disasters. To ensure feasibility, PCPs proposed (a) institutionalizing at-home vaccination in the organizational and operational scope of Greek PHC; (b) increasing and assigning the appropriate personnel for mobile SIV units; (c) securing appropriate vehicles and drivers; (d) and strengthening cooperation with municipal authorities (Table 3).

#### 3.2.8. Gamification

Though constituting a novel approach, most PCPs argued that utilizing video games to increase awareness regarding SIV was less effective in the Greek context. Gamification was not considered a feasible means of mobilizing patients to get vaccinated against the flu, as those in need of SIV were considered of low digital literacy and disinterested in learning and getting recommendations via this format.

Rasing awareness and promoting SIV through innovative video games was deemed valuable only if it targeted PCPs. The latter considered gamification to work better for increasing awareness of other vaccines targeting teenagers and young adults (Table 3).

## 4. Discussion

### 4.1. Main Findings

Our findings advance the existing literature on SIV by examining and providing the first qualitative insights on frontline PCPs’ attitudes and perceptions of SIV among adults in Greece. It further documents PCPs’ evaluation of strategies to enhance SIV uptake in the post-pandemic era. SIV was predominantly positively perceived by PCPs, who recognized its efficacy, safety and immense value in preventing and minimizing the severity of disease and complications. They lauded SIV’s acceptance among the general population and the subsequent ease in discussing and recommending SIV during patient–PCP consultations. In effort to increase SIV uptake, participants were in favor of utilizing all clinical encounters as opportunities to vaccinate and recommend SIV. They fully aligned with outsourcing vaccinations to allied health professionals under certain prerequisites, providing notifications to patients and PCPs alike about pending SIV, as well as with establishing at-home vaccination programs. Heterogeneity was recorded over the value and feasibility of adopting incentives targeting PCPs, with some lauding the necessary recognition of their work in prevention. Conversely, others noted that SIV is their professional duty and that provision of incentives would undermine confidence and value in PHC. The establishment of incentives targeting the general public was deemed an unsuitable strategy to increase confidence and uptake of SIV, as was promoting SIV in the eligible groups via gamification.

### 4.2. Comparison with Literature

#### 4.2.1. Attitudes Towards Seasonal Influenza Vaccination

In the post-pandemic landscape, SIV was overwhelmingly accepted and valued by participating PCPs. Indeed, SIV is widely accepted among PHC providers, with two thirds of German counterparts self-reporting favorable attitudes toward flu vaccination [25] and Spanish PCPs highlighting the importance of SIV for elder individuals [27]. Regional differences were noted in the German study, with PCPs practicing in regions with low SIV coverage reporting less favorable attitudes than providers in areas with high uptake [25]. Health workers in an Australian qualitative study underscored their confidence in SIV, despite variable personal beliefs regarding their disease complacency [41]. PCPs in our study additionally indicated that SIV is recognized among the general population. Indeed, Australian PCPs consider SIV to be widely accepted by patients as well, especially those belonging to high-risk groups [24]. In a similar manner, Spanish PCPs and nurses noted that patients over the age of 64 are informed and initiate and engage in discussions regarding SIV [27].

The safety profile of SIV, its effectiveness in preventing and mitigating severe illness, and its value in protecting vulnerable individuals were unanimously reported in our study and corroborated findings in the international literature [42]. SIV still constitutes the most effective intervention to prevent seasonal influenza [43]. Like Greek PCPs, health providers considered SIV to be imperative in minimizing the burden of disease on healthcare systems in a Jordanian study [44]. Echoing our PCPs’ beliefs, SIV curbs the work absenteeism and productivity loss [45], with Ferro et al. calculating the net benefit of flu vaccination for employees [46] and Maltezou et al. showcasing the protective effect of SIV in reducing absenteeism among healthcare workers [47]. Around four fifths of German and Indian PCPs also underscored the perceived effectiveness of SIV in preventing disease, as well as its reportedly few side effects and complications [25,48]. In a like manner, no serious adverse events following SIV were reported by Australian health workers [41], whereas Mexican providers considered SIV a safe practice for pregnant women [49].

#### 4.2.2. Views on Strategies to Strengthen Seasonal Influenza Vaccination

Participating PCPs commented on the strategies to increase SIV uptake and strengthen community coverage. Most highlighted the need to provide opportunistic counseling and recommend SIV at every encounter. Utilizing their long-standing relationship and trust with patients and their families, PCPs act as key informants and influence the former’s decision to undertake SIV [50]. Nearly 80% of German and Indian PCPs perceived patients’ decision to undertake SIV to depend on their sole recommendation [25,48]. However, despite previous studies in Australia and Greece highlighting the contribution of provider recommendation in citizens’ intention to receive SIV [11,51], less than half of Australian citizens noted having been recommended SIV in the past [51].

To avoid missed opportunities for SIV consultation and recommendation, the CDC recommends assessing patients’ immunization status during every clinical encounter [23]. Australian PCPs self-reported their capability to provide SIV consultation on an opportunistic and purposive basis in check-up appointments [24]. Checking patients’ status and counseling for SIV at every clinical encounter and during home visits are also considered important by nearly half and a third of PCPs respectively in a German study [25]. In a similar manner, 80% of Indian physicians noted that SIV should be a focal part in the patient–PCP agenda [48]. Though studied in pediatric patients [26], the effectiveness of cocooning strategies, i.e., prescribing SIV to chaperones and relatives, as proposed by few study participants, have yet to be examined in adult settings. Opportunistic recommendation for SIV is frequently hindered by increased workload, the complexity of presenting patient complaints and time pressure during consultations, a finding also stressed by Australian and Spanish PCPs [24,27].

Available evidence also suggests that outsourcing SIV to other health providers and local dispensaries is also deemed effective in increasing coverage. Increasing the number of personnel and locations in which SIV could be administered yields positive outcomes, provides numerous opportunities and facilitates access to vaccination for the general population [24]. Concerns were raised over the reduced impact of allied health workers on patients’ decisions to get vaccinated compared to PCPs [35]. However, the ‘Immunization neighborhood’, a collaborative approach between PHC units and pharmacies, strengthens outreach and highlights the pivotal role of allied PHC teams in increasing and documenting SIV uptake [28]. On the other hand, Australian PCPs highlighted the disruption in care a pharmacist-led SIV strategy poses [24], echoing Greek PCPs’ preference for SIV to remain under close physician monitoring. Reflecting the concerns of their Greek counterparts, American PCPs noted that pharmacists were not able to retract, understand patients’ medical history and vaccination status, and to record and communicate on-site vaccinations performed, strongly affirming their duty to monitor the former [28].

Healthcare provider and patient reminders, either automated through a digital platform or registry, or via physical means such as individuals’ vaccination cards, are deemed a gold standard of practice to enhance SIV uptake [23,29]. Indeed, their value in increasing coverage for seasonal influenza and other immunizations by as much as nearly 10% is highlighted in four systematic reviews [30,31,32,33,34]. Australian PCPs consider SIV recall systems to minimize missed SIV opportunities and enhance uptake, on condition that proper documentation on a respective registry is ensured [24]. Jacobson et al., in their systematic review, concluded that patient reminder systems aid in increasing uptake for all childhood, adolescent and adult vaccines, whereas Thomas et al. showcased that the former were also effective in boosting SIV uptake among individuals over the age of 60 [30,31]. With regard to adult immunization, the 2018 review indicated that the recipients of reminders were 1.29 times more likely to get SIV [31]. The provision of digital reminders and printed resources to patients aided Australian PCPs in bolstering SIV rates [24], as did the provision of invitations for SIV by French PCPs [52] and text messages provided by PHC practices in an American study [53]. Regarding the context and framing of patient reminders, the effect of personalized SIV invitations is underscored in a systematic review [30]. Ultimately, a study from Szilagyi et al. showcased no meaningful impact between loss-, gain-framed and commitment messaging [54].

Home visits as well as visits at long-term care facilities by PCPs and members of PHC teams provide a combined opportunity for vaccine and disease-related patient education, boost health literacy, and increase intention and vaccination rates [32,35]. Thomas et al. indicated that the recipients of home visits by health teams and PCPs had 1.3 times the odds of getting SIV [30]. Australian PCPs also expressed their interest in safeguarding vulnerable members of their community via administering SIV at home and long term care environments [24].

Participants were divided over the establishment of incentives for SIV targeting health providers and patients alike. Like their Greek counterparts, Australian health workers viewed SIV discussion and recommendation as key competences of their profession, as part of a duty of care towards their community [41]. Despite our participants’ calls to avoid commercializing core responsibilities of their discipline, providing financial remuneration has been proven to strengthen PCPs’ efforts in recommending and prescribing SIV. A past meta-analysis underscored that paid-for-performance PCPs had 2.22 times the odds for increased SIV uptake in older adults than their counterparts with pre-assigned, capitated payments [30]. Regarding the community, participants stood firmly against the provision of incentives, as these were perceived to undermine confidence in SIV. International evidence is inconclusive, with outcomes varying according to region, context and studied vaccine. Wong et al., in their review, noted the effectiveness of incentives in boosting COVID-19 vaccination rates [55], with Schwalbe et al. noting studies where cash transfers increased SIV uptake among older adults in Singapore and college students in the US [36]. Wang et al. noted that offering older adults financial incentives has a significant impact in undertaking SIV [34], as also reflected among younger age groups in another US study [37]. Reflecting our participants’ beliefs regarding the inability of incentives to promote confidence in vaccines, the former failed to sustain affirming SIV behaviors and uptake in the long-run amongst older Chinese individuals [56]. Provision of renumeration for SIV may intensify concerns over SIV, strengthen hesitancy, and may undermine immunization against the flu and other agents once respective incentives are removed [55].

The utilization of serious video games to increase SIV uptake was perceived as non-effective in the Greek context by study participants. However, this is widely viewed to boost awareness and address relevant issues regarding immunization. Two reviews mapped the available vaccination-related video games, with most addressing seasonal influenza [38,39]. Mostly targeting healthcare providers, they aimed to raise awareness regarding vaccination and respective guidelines. However, the absence of formal evaluation of gamification in vaccination promotion hinders their potential future use as an evidence-based strategy [39]. In a like manner, though immunization knowledge is improved, the impact of gamification in vaccine attitudes and uptake still remains to be further studied, as available evidence is contradictory [38,40].

Moving forward, within the organizational restrains and context of Greek PHC, the proposals by Greek PCPs in our study lay the groundwork for effective immunization strategies. Two recent reviews and meta-analyses underscored the importance of multicomponent interventions in increasing SIV rates [32,34]. Designing an effective SIV strategy should take into account, combine and implement PCP-valued motions to bolster community coverage. Opportunistic screening during all patient encounters is feasible, if the PHC workforce is aware of the value and is skilled in communicating vaccines. Strengthening communication skills training, providing tailored approaches, framing and patient-adapted recommendations empower PCPs to address patients’ concerns, increase vaccine confidence and uptake [57,58]. Upgrading the role of allied health workers within PHC units, strengthening coordination and working as a health team would also aid in reaching the set SIV targets. Allowing nurses and health visitors to triage and flag individuals in need for SIV minimizes missed opportunities, acting as a safety net for overburdened PCPs [24].

Following in the footsteps of successful reminder schemes, alert systems for health providers and patients alike are feasible and could potentially mobilize the community to consult health workers on immunization. However, to incorporate a successful reminder system for health providers and patients in routine care, proper and consistent documentation of past and performed immunizations in a respective registry is required [31]. Ensuring elongated consultation times with patients is also key in allowing PCPs to prioritize discussions, recommend SIV, reflect on and properly address patients’ concerns and proceed with an agreed immunization plan [35]. Nudging techniques could potentially increase vaccination behavior. Most notably, in an Australian study, health workers noted that simple triggers such as a reminder and the establishment of SIV hubs or trolleys at their work eased access to SIV and stimulated interest in getting vaccinated [41]. Flyers and posters in waiting rooms have yielded mixed results, with some studies attributing increased SIV rates to visual cues within the practice [30,59], whereas others noted no significant impact from this strategy [60].

The establishment of incentives toward health providers for ensuring their community’s vaccine coverage was considered a positive and effective measure, on condition that such remuneration is masked under the capitation system of the current “personal physician” payment scheme. Utilizing the lessons learnt from the COVID-19 mobile vaccination units and restructuring the organizational chart of Greek PHC to formally include home visits was a unanimous call in our findings. In addition, the establishment of mass vaccination events and clinics is viewed as effective in increasing SIV uptake in several international studies [24]. As proposed by two PCPs in our study, organizing mass vaccination events could increase the PHC unit’s ability and efficiency in providing SIV among the community, in cooperation with mobile vaccination units and local pharmacies. When designing and implementing mass events, care should be sought to safeguard patients’ privacy during SIV to increase satisfaction and retainment in the following season [24].

### 4.3. Limitations of the Study

The interpretation of our findings may be affected by specific limitations. To begin with, sampling though a professional organization and participants’ professional networks may have introduced selection bias in the study and therefore the participants cannot be considered representative of the PCP population in Greece. Vaccine enthusiasts were more likely to have responded to our open invitation and to refer like-minded colleagues as well. The final sample may thus have been homogenous regarding professional practices, attitudes and interest in immunizations. As such, opposing views and perceptions of non-responders and vaccine-hesitant PCPs may not have been captured in our findings. However, we utilized purposive and snowball sampling to recruit, gather and analyze the lived experience of a breadth of frontline PCPs with diverse demographic and professional characteristics across gender; work experience in PHC; and practice in urban, semi-urban and rural areas from 11 different prefectures in Greece. Indeed, dynamic and concurrent sampling, until coding and meaning saturation were reached, allowed us to capture in-depth insights on PCPs’ perspectives and proposals to strengthen SIV, with findings aligning and reflecting those of their international counterparts. Furthermore, the use of online platforms for conducting individual interviews may have deterred PCPs not familiar with the former from joining the study. However, the use of such platforms allowed us to invite participants from diverse regions and backgrounds to share their experiences with SIV in familiar environments, at home. Through this process, social desirability bias is considered to have been minimized, due to bracketing strategies, no prior acquaintance between the interviewer and the study participants, and the declaration of anonymization of transcripts and findings.

Our study reports on the attitudes of PCPs regarding SIV and their evaluation of strategies to increase confidence and the uptake of SIV among the general population. While PCPs’ perspectives are a vital piece of the puzzle and allow for the design of effective SIV campaigns and strategies, the attitudes of the public toward SIV in the post-pandemic era, and the appropriate strategies to enhance its uptake, also need to be examined in future cross-sectional studies. Furthermore, as our findings are deeply embedded in the specific socio-political and organizational framework of the Greek healthcare system, they may have limited transferability to other national contexts. While our findings provide an in-depth understanding of the phenomenon studied, future cross-sectional studies are warranted to determine the broader prevalence of these attitudes toward the evaluated strategies across a large representative sample of PCPs.

## 5. Conclusion

Frontline PCPs value the importance and effectiveness of SIV in protecting their communities. In the Greek primary care post-pandemic landscape, PCPs reported ease in discussing, consulting and recommending SIV during clinical encounters, as SIV is perceived to be widely accepted by the general population. In bolstering SIV uptake, findings indicate the significance of recommending SIV at all clinical encounters, delegating SIV to allied health providers and dispensaries, forwarding notifications regarding SIV to patients and PCPs alike, and establishing organized home vaccination programs. Although intended as tokens of appreciation, financial incentives for PCPs linked to strong SIV uptake may be perceived as potentially undermining confidence in and the perceived value of immunization efforts. The use of incentives for the general public may risk eroding trust and confidence in SIV and may be less suitable within the Greek context; similarly, gamification approaches to increasing SIV awareness may not be widely appropriate. PCPs’ insights are key in designing and successfully implementing strategies to increase uptake, coverage and confidence in SIV that are in line with community needs, values and lessons learnt from the COVID-19 pandemic. Within the organizational restrains of Greek PHC, effective policies are required to translate these qualitative insights into systemic improvements. Introducing SIV alert systems, providing incentives for health providers that reach SIV goals within their patient population and formally establishing at-home vaccinations in the PHC’s organizational chart are crucial in minimizing missed opportunities in SIV.

## Figures and Tables

**Table 1:** Demographic characteristics of participating Primary Care Physicians.

Participant ID	Gender	Years of Experience	Geographic Region	Area Status
1	Female	0–5	Western Greece	Rural
2	Female	5–15	Eastern Macedonia and Thrace	Rural
3	Male	0–5	Western Greece	Rural
4	Male	0–5	Attica	Urban
5	Female	15+	Attica	Semi-urban
6	Female	15+	Eastern Macedonia and Thrace	Rural
7	Female	0–5	Attica	Semi-urban
8	Male	0–5	Attica	Semi-urban
9	Male	5–15	Attica	Urban
10	Female	5–15	Central Macedonia	Urban
11	Female	5–15	Attica	Semi-urban
12	Male	5–15	Attica	Urban
13	Male	5–15	Thessaly	Urban
14	Male	15+	Central Macedonia	Urban
15	Female	5–15	Attica	Urban
16	Female	0–5	Central Macedonia	Urban
17	Male	5–15	Attica	Semi-urban
18	Male	15+	North Aegean	Rural
19	Female	15+	Central Macedonia	Rural
20	Female	5–15	Thessaly	Urban
21	Female	0–5	Thessaly	Rural
22	Male	15+	Western Macedonia	Urban
23	Female	15+	Western Macedonia	Rural
24	Female	0–5	Attica	Urban
25	Female	15+	Thessaly	Rural

**Table 2:** Primary Care Physicians’ attitudes towards Seasonal Influenza Vaccination presented through main themes and illustrative quotes.

Main Theme	Quotes	
Value	*We [health providers] love SIV as it keeps our community healthy.* P16 *SIV is a vaccine that may not be compulsory. Yet, most of us are living and working in environments where contact with frail and vulnerable individuals is common. It is important to get vaccinated for our own [health professionals] and their own [citizens] protection.* P8 *SIV also makes economic sense, as no working days are lost.* P8	
Effectiveness	*What I have noticed in the past 10 years that I have been getting SIV, is that I never got severely sick from the flu, compared to previous years when I wasn’t vaccinated. That’s what I noticed, and I think it is happening to my patients as well, at least those who are systematically vaccinated. Even drawing from personal experience, I think SIV is effective.* P6 *SIV aids in avoiding cramping in emergency departments and private practices. It reduces patient’s panic for medications and even antibiotics. This scenario repeats itself every year. SIV would aid in reducing visitation and workload in hospitals.* P14 *It is usually effective and protects extremely well, apart from few years when it fails to do so. The effectiveness of SIV in some seasons is drastically reduced. I think that two years ago, everyone contracted and got severely sick from the virus, whether vaccinated or not. This did not stop me from recommending it the following seasons though.* P11	
Safety	*I haven’t recorded any side effects. In the 25 years of performing SIV, there are no severe allergic reactions recorded, not even from those with reported egg allergy. We have of course some minor side effects in the injection site but nothing more serious. I do consider them safe.* P6 *SIV is an annually updated vaccine that has no side effects.* P7	
Acceptance	General population	*SIV is engraved in people’s minds. It constitutes the gold standard of immunizations.* P18 *We stopped discussing about SIV, because everyone gets vaccinated. It’s what we usually say, vaccines and especially SIV eventually undermined themselves because they were too effective and we stopped discussing about them. SIV is imperative.* P10 *From September to November, there is panic regarding SIV. People want to have a prescription, get to the dispensary, and get their vaccine. They are eager, they demand it themselves. Eighty to ninety percent of patients do this*. P20
	Health workers	*It is performed annually, it is important and it is at the top of the patient-physician agenda each season.* P5 *I recommend SIV and I think that for those over 60 years old, SIV should be mandatory, as they are the ones with greater complications from their respiratory system, should they contract the virus.* P7 *I would like to see SIV expanding to younger ages as well.* P14 *I do recommend SIV to all patients, and not necessarily to those with risk factors. I think that universal vaccination helps. If a younger patient reaches out and asks for SIV, I won’t tell them that this is not recommended for them.* P21

**Table 3:** Views of PCPs toward strategies to strengthen Seasonal Influenza Vaccination, presented through main themes on the value and challenges of each strategy (where applicable) and illustrative quotes.

Strategy	Themes on Value and Challenges of Each Strategy	Quotes
Opportunistic counseling regarding SIV at all clinical encounters	**Value** - Duty of care - Vaccination of chaperones - Prerequisites (lengthier appointments, patients’ health literacy, trust in PCPs)	*It’s what we ought to do. It’s our duty as health providers in PHC to approach the family and have them all in mind during a clinical encounter. “Are you vaccinated?” It’s a simple question.* P13 *It is the PCP’s duty to take note of the family and take care of the patient’s family as well. I only see [opportunistic counseling to relatives] as something good. One’s protection may contribute a lot to others as well.* P22 *If you have time and the clinical context allows you to do so, then, yes, suggest SIV at all times.* P1
	**Challenges** - Lack of time - Chaperones’ resistance - Need to avoid conflict	*It’s difficult to also take time to talk with chaperones, who might have their own issues and need a different approach. There is no time to think about it and do it.* P16 *It would be much harder to convince them. Most are convinced of the need to vaccinate high-risk groups, yet are reluctant when asked about themselves.* P7 *I sometimes get too tired when discussing SIV with one individual, let alone two. You get it, right? It is too difficult for me, and I don’t have the time, the strength and the willingness to do so.* P18
Outsourcing vaccination to nursing, allied health workers and community pharmacists	**Value** - Empowering non-physician health workers - Prerequisites (access in the SIV registry, rigorous training, SIV eligibility assessment, overview by PCPs)	*We [primary care professionals] are all in this together, as partners, as team, we all try our best to increase coverage for our community.* P6 *Community pharmacies in Greece are like a secondary PHC system. Since vaccines are out there, what are we so afraid of? Nothing at all! Let the pharmacists perform SIV, so long as the SIV registry is updated.* P12 *I am fine, so long as the performed SIV is recorded in a registry, and I can access and see what patients have done.* P22 *They should be trained and should know when to perform SIV, if there is need for simultaneous vaccinations with other agents as well. We should be properly trained and perform vaccinations in sites where complications can be addressed.* P6 *Someone could check whether the criteria set by the National Immunization Program are met, and whether an individual fits the prerequisites*. P1 *PCPs should be the main orchestrators. You can of course get SIV at other sites and with other providers but never lose contact with a physician.* P3
	**Challenges** - Care responsibility by PCPs - Errors in past pharmacy vaccinations	*I wouldn’t like it as it creates a sense of insecurity. If a patient has consulted me and we are only talking about the act of SIV, then I am fine. But performing vaccination without physician’s approval seems a bit odd for me and I wouldn’t want this. It is a medical procedure, a therapeutic, a preventive one that no one else can perform.* P25
Notifications regarding SIV to the general population	**Value** - Strengthening awareness of immunizations - Empowering control on health - Broadening the scope of existing notification schemes - Prerequisites (personalized content, strong political will, cues to contact their PCPs)	*It will work for some individuals. Sending notifications will mobilize them and they will visit and discuss SIV with us or a pharmacist.* P4 *Just like the rest of the screening programs, citizens will positively respond to notifications regarding SIV. It would be valuable for them to receive texts reminding them to visit PCPs.* P18 *It’s a matter of political will. The Ministry has reminded virtually everyone about mammography and colon cancer screening. Such reminders are proven to be easy to send and successful in bringing citizens to pharmacies and physicians.* P3
	**Challenges** - Low levels of digital literacy - Exclusion of older individuals - Perceived ineffectiveness of past notifications	*When health literacy is non-existent, then people view doctors as the professionals to visit in need. If we want to challenge this perception and promote our health and well-being, then we need reminders in this new, holistic context. It is important to know, to be reminded, to be motivated to contact PCPs. It creates a healthy culture, supporting well-being and health promotion.* P8 *If PCPs or pharmacists are not aware and are not actively asking patients whether they received a SIV-related text, nothing will work. Unless PCPs mobilize patients, no SMS would mobilize them to get vaccinated. Individuals have no willingness to proceed with vaccination, unless a PCP reminds them to do so. They will read the SMS and delete it or archive it.* P6
Notifications regarding SIV to PCPs	**Value** - Facilitating PCPs’ work - Safety net regarding immunizations - Prerequisites (establishment of vaccine registry, training of PCPs, lengthier appointments, appropriate placement of notification on digital platforms)	*I find it useful to get a notification in the prescription platform when registering patients. In this way, through discussion of pros and cons and its value, you can remind patients to get SIV.* P20 *Sending notifications would be useful, as SIV might not always be in our mind. We wouldn’t have to actively search each individual’s electronic health record and see whether it’s missing for this season. We don’t always have time… in this case, those not familiar and aware of the value of SIV would get a valuable reminder and discuss something that would otherwise not be part of the consultation agenda.* P4
Adoption of incentives for the general population	**Value** - Deductions in medication and laboratory examinations - Co-payment of hospitalization costs	*Patients with fully updated vaccinations are less costly for healthcare systems. In this context you may offer those covered for SIV deductions in medicines and lab exams to positively reinforce vaccination.* **P4** *We shouldn’t cover the costs of hospitalization for unvaccinated patients. If citizens chose not to get SIV for their own reasons, the health system should not burden with the cost of their hospitalization. Co-payment is a great way to boost SIV rates.* P11
	**Challenges** - Minimizing the public’s confidence in SIV - Strengthening conspiracy theories - Spill over SIV hesitancy in childhood vaccines	*I could be useful, but we would lose the point. We have to change people’s mentality about primary prevention. Greeks are not aware of primary prevention and should we bring it down to merely providing incentives for SIV, we would lose its meaning. They won’t get SIV because they understood its importance. We would increase uptake, but we would lose the meaning, the public’s faith in vaccines as primary prevention mechanism.* P7 *Providing incentives will only heat up conspiracy theories. Citizens would wonder: ‘why are doing this? They are hiding something. Why would they pay us otherwise?’. I think this would cancel and reverse all PCPs’ efforts to convince them that they do the best for their health. The Greek culture is against paying Greeks to do things that promote their health and wellbeing. It would most likely backlash.* P6
Adoption of incentives for PCPs	**Value** - Recognition of PCPs’ work - Motivating PCPs	*I tell them to get vaccinated. Others don’t, other PCPs won’t care. Vaccines won’t be something that patients ask for or complain about when not being recommended one. So, it is great to have incentives for those of us that go the extra mile each time.* P3 *We could introduce an incentive based on how many patients one has vaccinated against the flu. That could motivate PCPs. I would be more open to this solution.* P5
	**Challenges** - SIV is PCPs’ duty - Minimizing the public’s trust and confidence	*Vaccination is part of our work and should be in every PCP’s agenda. I don’t believe in any motives, as it’s a core duty and we should all have it in mind at work.* P21 *Providing incentives to PCPs will have the public thinking that we commercialize medicine. They will think that we get paid to offer them SIV and they would not positively regard this.* P24
Establishment of at-home vaccination programs	**Value** - Protection of susceptible citizens - Outbreak prevention - Prerequisite (formal integration within PHC’s scope, specialized personnel, cooperation with local authorities)	*The COVID-19 pandemic allowed us to see how this could work in our country. We definitely need more personnel to orderly vaccinate year-round, but we saw that at-home vaccinations are feasible and they can only bear fruitful results. If only it can be incorporated in the organizational structure of PHC and be of binding nature so that all PHC could offer at-home SIV in the future.* P25 *It is extremely important for vulnerable individuals that reside together and a potential domino effect can be averted. It is also extremely important for vaccinations to be performed by local PHC units’ workforce. SIV is a weapon of mass protection and must be widely used.* P12 *There needs to be an organized framework for at-home and mobile vaccination programs. We shouldn’t rely on the goodwill of individual PHC units.* P23
Gamification	**Value** - Strengthening PCPs’ awareness - Effective in vaccines for teenagers and young adults	*Maybe for the youth and HPV vaccination. Not for SIV and in older ages for sure.* P1 *The whole point is for gamification to target physicians, pharmacists, health visitors and everyone that vaccinates. It would help us learn and increase awareness in a fun way. It should target us, who are supposedly better accustomed to digital media, and not the general public.* P13
	**Challenges** - Digital literacy - Disinterest in digital format	*Those we need to vaccinate, the elderly, have no ability to manage and comprehend such technologies. There would be no point in doing that. We would vaccinate more against the flu by getting out in the community and spreading the news than utilizing games which are available 24/7. One must be acquainted for such platforms to work.* P20 *For adults? No way! It may work at a research level in Greece for the time being. The public is not ready to support such initiatives.* P3

## Data Availability

The data presented in this study are available on request from the corresponding author.

## Supplementary Materials

The following supporting information can be downloaded at https://www.mdpi.com/article/10.3390/vaccines14050458/s1.
