# Supplementary material for: Strategies to Enhance Seasonal Influenza Vaccination Uptake: Qualitative Insights from Primary Care Physicians in Greece
Source: Vaccines (Basel). 2026 May 20;14(5):458. doi: 10.3390/vaccines14050458 (PMC13211693; doi:10.3390/vaccines14050458)
Supplement: Supplementary file 1 [file vaccines-14-00458-s001.zip › vaccines-4272741-supplementary.pdf]

Supplementary file S1. The part of the interview guide relevant to the findings of this article.

1. Please, share your thoughts and comments on seasonal influenza vaccination (SIV).

2. How can we best increase the uptake and confidence in seasonal influenza vaccination?

3. Please share your thoughts and comments on the feasibility and effectiveness of the following strategies to enhance seasonal influenza vaccination, as reported in literature:

[Exclude those mentioned in question 2. Include any novel proposed strategy from participants to the list and explore its feasibility and effectiveness with future study participants.]

(a) Opportunistic SIV at every encounter;
(b) SIV by non-physician healthcare workers;
(c) Reminders to the general population regarding SIV;
(d) Reminders to health workers regarding SIV;
(e) At-home SIV programs;
(f) Establishing incentives for health workers;
(g) Establishing incentives for the general population;
(h) Gamification;
